# 

**Published:** 1921-05-28

**Authors:** 


### Points from Annual Reports.

### National Hospital for the Paralysed and Epileptic.

The financial position was so unpromising that in June 1920 the hospital ceased to receive any more in-patients; in July it was decided to close three wards only; in October the whole hospital was opened once more. This happy result was largely brought about by an emergency grant of £8,000 from the King's Fund and increases in revenue all round. A survey of the accounts shows that more regular support is required before the hospital can be said to be "out of the wood."

### Essex County Hospital.

The hundred and first annual report opens with a note of regret that the waiting-list of patients has increased, and it continues with an expression of anxiety for the future of the institution. There was a deficit of £5,810 last year, and to reduce expense the report consists of only twelve pages.

### St. George's Hospital.

The Committee call special attention to the fact that the expenditure has increased from £40,358 in 1913 to £85,626 in 1921. Additional income of at least £30,000 per annum must be secured. The ordinary expenditure exceeded the ordinary income by £35,753 last year, and although legacies amounted to £12,842, an emergency grant of £8,500 was received from the King's Fund and £8,850 from the National Relief Fund, there still remained a deficit of £6,631.

### Royal Sea-Bathing Hospital, Margate.

In spite of considerable labour troubles, the King George V. Wing has been completed, and sixty-eight beds have been added to the accommodation of the hospital. The total cost of the wing, with equipment, was £19,161, and towards this the Ministry of Health made a grant of £10,380. The total income for the past year was £36,379, and the total expenditure £35,087. The average cost per occupied bed was £144 11s. 5d. The institution received for patients under the National Health Acts the sum of £19,305.

### Personalia.

Mr. William Briggs, J.P., has been elected President of the Clayton Hospital, Wakefield. Mr. Briggs, who belongs to an old Wakefield family, has held office as a Vice-President for many years, and has been associated with Mr. Tew, the late Chairman, in many matters affecting the interests of the hospital. It is interesting to record that at the time the present hospital was being built the contractor failed, and the work was carried to its conclusion by the father of the new President.

### PASSING EVENTS AND TOPICS.

### Accommodation for Consumptives at Army Camp.

Plymouth Corporation has prepared plans for the provision of accommodation for two hundred cases of tuberculosis at the Efford Camp, but the Health Ministry notifies that it does not feel justified at the moment in sanctioning such an extensive scheme. It will approve as an instalment to the complete scheme proposals for the immediate provision of not exceeding forty hospital beds at the Camp, and thirty beds for surgical cases. The Health Committee of the Corporation points out that the treatment requires the supervision of a skilled surgeon of experience in the modern methods of treating surgical tuberculosis and the provision of expensive equipment and appliances. It would, therefore, be uneconomical to provide a small institution. The Committee has decided to send a deputation to the Ministry to discuss the matter.

### A North Riding Benefactor.

Mrs. Edward Shaw, O.B.E., Commandant of the Ryedale and District Red Cross Detachment, has made a magnificent gift to the North Riding of convalescent homes for Army pensioners. The homes, which are situated near Kirbymoorside, consist of a group of four converted Army huts, which provide accommodation for forty beds, with recreation and billiard rooms, and excellent staff quarters. They were opened on April 7 by Major Bower, C.M.G., C.B.E., County Director of the Red Cross, who spoke warmly of Mrs. Shaw's generosity and unflagging work for the benefit of the disabled and of the youth of the North Riding.

### Hospital President Retires.

Major Spencer Chichester, for many years President of the Romsey Nursing Home and Cottage Hospital, has now retired and is leaving the district. A tablet recording the services of himself and Mrs. Chichester is to be placed in the hospital, which in consequence of special efforts last year is now in a stronger position than it has been for some time. Major Chichester can therefore look back on his term of office with much satisfaction, and the institution under his successor should develop on its new lines.

### Post-Graduate Lectures.

A Course of Clinical Lectures and Demonstrations will be given at the Queen's Hospital for Children, Hackney, during this and the ensuing month. The lectures will be free to all medical practitioners and students of medicine on presentation of their visiting card. Mr. Kenneth A. Lees, 48 Harley Street, W. 1, the Hon. Secretary to the Medical Committee, will be glad to supply any further particulars.

### The London Hospital's Catgut Industry.

The catgut department of the London Hospital, which produces the needs of other institutions as well as its own, announces a system, after nearly a year's investigation, which makes it possible to produce in large quantities a surgical catgut which is completely aseptic.

### Pharmaceutical Society's New Policy.

At the annual meeting of the Pharmaceutical Society of Great Britain, held on May 18, the President made an interesting reference to the policy now to be adopted, consequent on the decision of the High Court, which ruled that the Society cannot legally deal with "trade" matters. For the future the Pharmaceutical Society will give its attention to the academic and professional side of pharmacy, including examination and registration duties. It is proposed to resume the professional post-graduate lectures next winter, as in pre-war times. Institutional pharmacists will without a doubt welcome the new policy, which should do much to develop public pharmacy on professional lines. Pharmacists have long been exercised over the absence of a legal statutory qualification to be held by those who dispense medicines at hospitals and other public institutions. As the law stands, anyone may assume the title of "dispenser" and act in that capacity. This is a matter to which the reformed Pharmaceutical Society might well give careful and constructive attention.

### Central Midwives Board for Scotland.

The examination of the Board on May 2 and 3, held simultaneously in Edinburgh, Glasgow, and Dundee, has concluded, with the following results: Edinburgh, 49 candidates, 45 passed; Glasgow, 86 candidates, 79 passed; Dundee, 14 candidates, 12 passed.

The following were the questions; the time given was three hours:

(1) What are the uses of the liquor amnii in pregnancy and labour? Describe the effects of hydramnios on labour and its dangers to the mother and child. (2) What are the chief danger signals for which a midwife ought to be on the outlook during the first four months of pregnancy? Describe the treatment of any two of them. (3) Give the diagnosis and management of a breech presentation. What are the dangers (*a*) to mother and (*b*) to child? (4) In what circumstances is it necessary to catheterise a patient? Describe in detail the method of passing the instrument. (5) What are the signs of prematurity in the new-born child? What are the difficulties which may be encountered in the management of a premature child? How should these be dealt with? (6) What do you consider to be meant by "serious skin eruptions" in the Rules of the Central Midwives Board, and what is your duty with regard to them?

Certificates Cancelled.

At a meeting of the Board for the hearing of penal cases, Dr. J. Haig Ferguson in the chair, No. 2,540, Jane Nugent, Lanarkshire, was cited to answer charges of failure to send for medical assistance in the case of a patient suffering from post-partum hæmorrhage with raised temperature, and with failure to take and record the pulse and temperature of her patients and to keep her register of cases, and with other breaches of the Rules. The Board found the charges to be proved, and instructed the Secretary to remove the name of Jane Nugent from the Roll of Midwives and to cancel her certificate, and further, in terms of Section 8 of the Act, she was prohibited hereafter from attending women in child-birth in any other capacity.

At the same sitting the case of No. 1,773, Mary Nicol Martin, Kelty, which had been adjourned for judgment on report of the Local Supervising Authority (Fife County), was under consideration. The further report on the methods of practice of Mary Nicol Martin being unfavourable, the Secretary was directed to remove her name from the Roll of Midwives and to cancel her certificate.

### The Living-out Question.

RECOMMENDATIONS FOR M.A.B. MENTAL STAFFS.

The London District Council of the National Asylum Workers' Union has asked, on account of the dissatisfaction expressed by many of the M.A.B. staffs with the accommodation provided, and the charge made, that the Board will permit all members of the staffs who so desire to board and lodge out of the institution. The Mental Hospitals Committee of the Asylums Board has gone into the matter and reported. The present position is that the staff are allowed to live out whenever practicable. At present a very large number of the staff, both male and female, of the mental hospitals actually do live out. At Leavesden the proportion is much greater than elsewhere. At Tooting all the male staff live out, and at Darenth and Caterham there is a fair proportion of non-resident staff. But the majority of non-resident staff board in the institution, and for this they pay at the rate of 2s. 4d. per day for full board. As the staff state they are dissatisfied, apparently this arrangement is not regarded as advantageous by them, and the Committee is of opinion that it is not advantageous to the Board, because, apart from the amount of book-keeping and clerical work involved, a large staff is employed in the kitchens and elsewhere in the provision and serving of meals. Hence the Committee has come to the conclusion that (*a*) all accommodation originally provided for resident staff should be utilised before staff are allowed to live out. The Committee does not insist that accommodation should be utilised by the staff, but it considers that it ought not to be allowed to remain vacant considering its original cost to the Board. So long, however, as it is made use of for some good purpose, the Committee considers that it does not matter whether it is occupied by staff or in any other way. (*b*) Conversely, the Committee thinks that the patients' accommodation, which at some institutions is now being used for resident staff, ought to be freed with the least possible delay, as the beds are needed for their proper purpose, and while they are used for staff they represent a serious loss to the Board; (*c*) that as an experiment non-resident staff should be required to provide their own meals, rooms being set apart in which they may partake of them.

### Gifts and Bequests.

Mr. J. E. Abrahams, of Maida Vale, has left £50 each to nine Melbourne hospitals.

The Royal Victoria Hospital, Belfast, has been left £250 under the will of Mr. Andrew Henderson, of Whiteabbey, Antrim.

The Royal Surrey County, Guildford, has received £6,500 under the will of Mr. Peter John Crooke, of Priory Road, Kew.

Mr. A. H. Siddons, of West Bromwich, has left £50 each to West Bromwich District Hospital and the District Nursing Association.

The Royal Midland Counties Home for Incurables, Leamington, will receive £200 under the will of Vice-Admiral George Northland.

Subject to certain life interests, Mr. F. N. Andrews, of Melton Mowbray, has left part of the residue of his estate to Melton Mowbray Cottage Hospital.

Mr. George Radford, of Church Stretton, has left £200 to the Hospital for Incurables, Mauldreth, and £150 to the Manchester Hospital for Skin Diseases.

Birkenhead Borough Hospital will receive £1,000 and Wirral Hospital for Sick Children £200 under the will of the late Mrs. Ellen Fawcett Laird, of Birkenhead.

Mr. William Burkitt, of King's Lynn, has left £1,000 to the Chesterfield and North Derbyshire Hospital, and £1,000 to the King's Lynn and West Norfolk Hospital.

A sum of £2,000, together with a gift of appliances and equipment to the value of over £300, has been received by the County Infirmary, Londonderry, from the Londonderry County Red Cross Funds.

Princess Christian has sent £200 for the funds of Egham Nursing Association, of which she is President. The money is part of the proceeds of the sale of the Princess Christian Military Hospital at Englefield Green.

Mr. Alfred Fairley, of Handsworth, Birmingham, has left £1,500 to the Birmingham General Hospital to endow a bed primarily for the use of Freemasons or their wives and families, and £250 to the Birmingham General Dispensary. The General Hospital will also receive £200 under the will of Mr. John Fawdry, of Hastings.

Mr. J. T. Dorrington, of Wallsworth Hall, Gloucester, has left, after the death of his wife, £1,000 each to the Manchester Eye Hospital, the Gloucester General Infirmary, and the Free Hospital for Children, Kingsholm, Gloucester, and £500 each to the Manchester Cancer Pavilion and St. Mary's Hospital for Women and Children, Manchester.

Mr. R. F. Samuels, of Liverpool, has left £1,500 to the University of Liverpool for three scholarships in medicine, surgery, and obstetrics and gynæcology, £200 to the Liverpool Royal Infirmary, £100 to the Queen Victoria Nursing Association, £200 each to the Hospital for Women, Liverpool, David Lewis Hospital, the Liverpool Southern Hospital, and the Stanley Hospital.

THE
### COLLEGE OF NURSING
(Limited by Guarantee.)

### BIRMINGHAM THREE COUNTIES CENTRE.
(Hon. Secretary, Mrs. Glegg, 84 Hagley Road.)

The Scenic Fair.

On June 2 to 11 a nine days' Fair will be held in Bingley Hall, Birmingham, to place the Birmingham and Three Counties Centre of the College of Nursing on a self-supporting basis, and to establish a Club for all trained nurses in the Three Counties. The Fair will be opened by the Lady Mayoress of Birmingham (Mrs. W. A. Cadbury), and on June 6 H.R.H. Princess Mary's visit will be paid. Presentations will be made to the Princess, who will make a tour of the stalls, receive purses, and take tea in the Fair. The Hon. Sir Arthur Stanley, Chairman of the College of Nursing, will be present on June 6 and 7, and on the latter date Lady Cowdray is coming specially to Birmingham to visit the Fair. On June 2, 6, and 7 nurses in the Three Counties will form a Guard of Honour. Any nurse wishing to stand in the "Guard of Honour" must report in indoor uniform at Bingley Hall not later than 2.15 p.m. on the days mentioned.

### NORTHUMBERLAND AND DURHAM CENTRE, NEWCASTLE-ON-TYNE.
(Secretary, Miss Toyne, 17 Windsor Terrace, Jesmond.)

Friday, June 3, 6.30 p.m.—Members' meeting at the Nurses' Club, 17 Windsor Terrace. The question (which has already been under discussion) of admitting other professional women as well as nurses as members of the Club will be decided. It is therefore hoped all members will attend.

Annual subscriptions for the Centre and the Club became due on April 1. The Secretary will be glad to receive them as soon as possible.

### SWANSEA CENTRE.
(Hon. Secretary, Mrs. F. W. Jenner, Glynn Vivian Home, Mumbles.)

A club-room has been opened at the Y.M.C.A. House, St. Helen's Road, Swansea. It will be open daily from 10.30 a.m. to 7 p.m., and will serve as temporary headquarters for the Centre.

### Forthcoming Events.

The League of Remembrance (1914-1919), whose headquarters are at 1 Marlborough Gate, W., will hold its first anniversary dinner at the Connaught Rooms on Tuesday, June 28, 1921, at 8 p.m. H.R.H. Princess Beatrice and H.R.H. the Duchess of Albany have consented to be present, and Major Beith (Ian Hay) and Mrs. H. B. Irving have promised to speak. Tickets, the cost of which is 12*s.* 6*d.* each (exclusive of wine), can be obtained from the Hon. Secretary, 1 Marlborough Gate, W. 2.

### Matrons' and Sisters' Appointments.

Royal Berkshire Hospital, Institution for Private Nurses, Reading.—Miss A. B. Barter has been appointed lady superintendent. She was trained at St. Thomas's Hospital, has been assistant matron at the Swansea General Hospital, matron of the Coventry and Warwickshire Hospital, lady superintendent of the Fakenham Nurses' Home, and matron of Manchester Royal Eye Hospital.

London Jewish Hospital, Stepney Green.—Miss Jean Todd has been appointed out-patient sister. She was trained at the Royal Infirmary, Sheffield, where she was later out-patient theatre sister; she has been theatre sister at the Bradford Royal Eye and Ear Infirmary, and out-patient sister at the Royal Chest Hospital, City Road.

Metropolitan Hospital, E.—Miss Ina Docherty has been appointed night sister. She was trained at the Dumfries and Galloway Royal Infirmary, has been staff nurse at Edinburgh Royal Infirmary, theatre sister at Stanley Hospital, Liverpool, ward sister at Dumfries Royal Infirmary, night sister and acting matron at Great Yarmouth General Hospital, and matron of the County Infirmary, Brecon.

Gloucester Royal Infirmary.—Miss Florence E. Burrows has been appointed sister. She was trained at the North Devon Infirmary, Barnstaple, has been staff nurse at the Coventry and Warwickshire Hospital, and sister under the British Red Cross Society. She is a member of the College of Nursing.

Birmingham and Midland Ear and Throat Hospital.—Miss J. M. Symonds has been appointed matron. She was trained at St. Bartholomew's and the East London Children's Hospitals; she has been assistant home sister at the former institution, and assistant matron at the South Devon and East Cornwall Hospital.

City of Westminster Union Infirmary, Fulham Road.—Miss Jennie Willens has been appointed first assistant matron. She was trained at the Portsmouth Union Infirmary, where she was later sister and maternity sister; she has been night sister and home sister at Burnley Union Infirmary, assistant superintendent Ashton-under-Lyne Union, and home sister and night sister at Isleworth Infirmary. During the war she was in Italy under the T.F.N.S. as staff nurse, sister and assistant matron, and later home sister at the Ministry of Pensions Hospital, Orpington. Miss Hilda Emma Mills has been appointed second assistant matron. She was trained at Leicester Royal Infirmary, where she was later sister of various wards and departments; she has also been night superintendent of Salop Royal Infirmary, sister and night superintendent of the 5th Northern General Hospital, Leicester, and home sister and tutor-sister at the Bradford Royal Infirmary.

Children's Sanatorium, Morris Grange, near Richmond, Yorks.—Miss Elizabeth Jackson has been appointed matron. She was trained at the General Hospital, Nottingham.

Staffordshire Mental Hospital, Cheddleton, Leek.—Miss L. Haxell has been appointed assistant matron. She was trained at the Middlesex Hospital and the Hospital for Sick Children, Great Ormond Street. She has been ward sister at the General Hospital, Birmingham, sister-in-charge of the infectious block, and ward sister at the Hospital for Sick Children, and served in France for some years as sister in Q.A.I.M.N.S.R.

City Mental Hospital, Gosforth, Newcastle-on-Tyne.—Miss Alice Nixon has been appointed assistant matron. She was trained at the Hartlepools General Hospital, where she was later night sister; she has been staff nurse and night superintendent at Northumberland War Hospital, Gosforth, and assistant matron at James Murray's Mental Hospital, Perth.

Dudley Union Infirmary.—Miss Edith E. Brown has been appointed sister. She was trained at Sheffield Union Infirmary, where she was later staff nurse.

May 28, 1921] [Price Twopence

### The Hospital

**RATES OF SUBSCRIPTION** (post free, payable in advance).
Three months, 3/3; six months, 6/6; twelve months, 13/-. (Should the cost of printing and paper as well as increased postage, necessitate alteration of these rates, the period paid for will be reduced proportionately on unexpired subscriptions.) THE HOSPITAL "Index" to Volume LXIX., October 1920 to March 1921, is now ready, price 1/1 per copy, post free. Address The Manager, THE HOSPITAL, "The Hospital" Building, 28/29 Southampton Street, Strand, London, W.C. 2.

